# Sample Volume Reduction Using the Schwarzschild Objective for a Circular Dichroism Spectrophotometer and an Application to the Structural Analysis of Lysine-36 Trimethylated Histone H3 Protein

**DOI:** 10.3390/molecules23112865

**Published:** 2018-11-02

**Authors:** Yudai Izumi, Koichi Matsuo

**Affiliations:** Hiroshima Synchrotron Radiation Center, Hiroshima University, 2-313 Kagamiyama, Higashi-Hiroshima, Hiroshima 739-0046, Japan; pika@hiroshima-u.ac.jp

**Keywords:** Schwarzschild objective, beam focusing, histone, post-translational modification, structural alteration

## Abstract

With the increasing interest in scarce proteins, reducing the sample volume for circular dichroism (CD) spectroscopy has become desirable. Demagnification of the incident beam size is required to reduce the sample volume for CD spectroscopy detecting transmitted light passed through the sample. In this study, the beam size was demagnified using a focal mirror, and small-capacity sample cells were developed in an attempt to reduce the sample volume. The original beam size was 6 × 6 mm^2^; we successfully converged it to a size of 25 × 25 μm^2^ using the Schwarzschild objective (SO). The new sample cell and SO allowed the required sample volume to be reduced to 1/10 (15 → 1.5 μL), when using a 15 μm path length cell. By adopting a smaller sample cell, further sample reduction could be achieved. By using the SO system, the secondary structural contents of the lysine-36 trimethylated histone H3 protein were analyzed. The trimethylation induced the increment of helix structures and decrement of unordered structures. These structural alterations may play a role in regulating cellular function(s), such as DNA damage repair processes.

## 1. Introduction

Circular dichroism (CD) spectroscopy in the ultraviolet (UV) region is widely used for the secondary structural analysis of proteins in an aqueous solution. Although the structural information from CD spectra is limited compared with that from X-ray crystallography and nuclear magnetic resonance (NMR), both of which display three-dimensional structures with atomic-level resolutions, CD spectroscopy is a powerful tool because it can more easily provide structural information, including the structural dynamics, because of some notable advantages: (i) the required sample amount is smaller (1–10% of those needed for X-ray crystallography and NMR [1]) and (ii) the samples can be easily prepared by simply dissolving the proteins in a solvent. Neither crystallization nor isotopic substitution is required. Therefore, the loss of the samples and accidental denaturations during sample preparation are negligible in most cases.

The use of synchrotron radiation (SR) as a light source for CD spectroscopy allows more precise structural information to be obtained, because an SR beam can expand the measurement region to the vacuum-ultraviolet (VUV) region where additional CD peaks are often observed. Indeed, synchrotron radiation circular dichroism (SRCD) spectroscopy has produced successful outcomes over the past two decades [2]. It has also become desirable to reduce the sample volume with the increasing interest in scarce proteins that are difficult to synthesize.

Although the sample volume could be reduced using small-capacity cuvettes, this is insufficient in most cases of CD spectroscopy. Most commercial and SR-based CD spectrophotometers adopt a so-called “transmission method”, i.e., they detect transmitted light passed through the sample and provide CD spectra. In this case, the required sample volume largely depends on the size of the incident beam because the sample area must be larger than the beam size for the whole beam to pass through the sample. Therefore, most CD spectrophotometers require beam-size demagnification and cuvette capacity reduction.

One of the better ways to demagnify the beam size is using lenses and/or mirrors to focus the beam. Indeed, some groups have succeeded in measuring CD spectra using focal beams (although their motivations were different from that of this study). For example, Kane et al. focused the beam on a spot size of 20 × 60 μm^2^ using a focal lens and succeeded in measuring the CD spectra of a cytochrome *c* protein using a micro flow channel of 200–250 nm at the German SR facility BESSY II [3]. Yamada et al. developed a circularly polarizing microscope using the Schwarzschild objective (SO), combined with a convex mirror and polarizing undulator, at the Japanese SR facility TERAS. They achieved a sub-micron beam (0.66 μm at wavelength 200 nm) and obtained a CD image of a *d*-10-camphorsulfonic acid film on a copper grid [4]. For CD spectroscopy, the focusing systems must conserve the polarization degree, and chromatic aberrations must be eliminated in the wide wavelength range. The use of focal lenses is generally not enough to eliminate aberrations for wide-range CD measurements in the UV region to the VUV region. However, according to Yamada’s complicated system, it seems that the chromatic aberration due to the SO systems is negligible or small. Thus, for this study, the SO was installed on a VUV-CD spectrophotometer, which uses the SR light of beamline BL-12 [5] of the Hiroshima Synchrotron Radiation Center (HiSOR), to demagnify the incident beam size. Small-capacity sample cells were also developed. As a result, the sample volume for CD spectroscopy was successfully reduced using the SO and cells. In addition, a CD spectrum of a scarce protein, lysine-36 trimethylated histone H3 protein (H3K36me3), was measured using the same system, and its secondary structural contents were analyzed.

In the next section, the performance of the new VUV-CD spectrophotometer measurement system and the application of this system to the structural analysis of H3K36me3 are reported.

## 2. Results and Discussion

### 2.1. Beam Focusing by the SO

**Figure 1:**
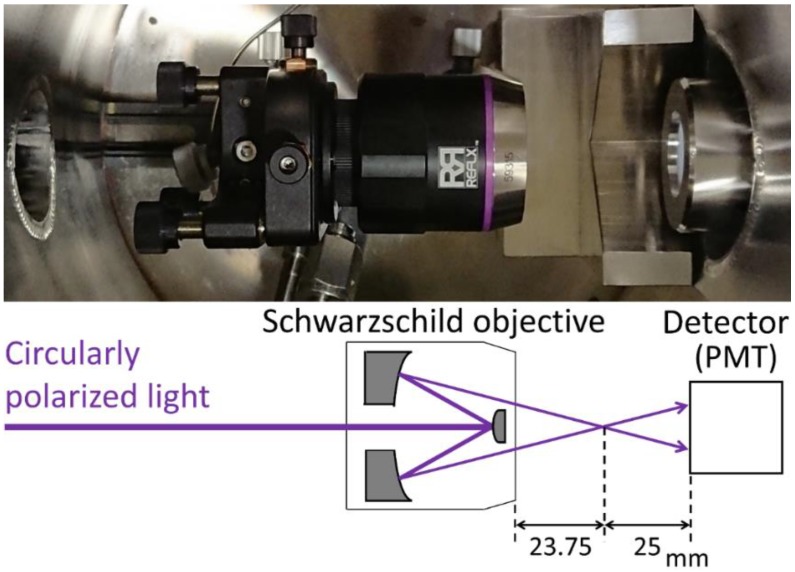
(**Top**) Top-view photograph of the VUVCD spectrophotometer sample chamber after Schwarzschild objective (SO) installation. (**Bottom**) A schematic view of beam focusing using the SO.

The SO (Infinite Conjugate, DUV Coated, 15X/0.28NA, ReflX Objective; Edmund Optics, Barrington, NJ, USA), which had a working distance of 23.75 mm, was installed in front of the detector (photomultiplier tube: PMT) (Figure 1). Since the SO consisted of two spherical mirrors with coincident curvature centers, the incident beam was reflected twice and then focused. After that, the beam spread out for the detector. The position of the SO was precisely adjusted using a mechanical mount (5-axis kinematic mount; Thorlabs, Newton, NJ, USA) as the beam passed through the center of the sample and detector. The distance between the focal position and the detector was about 25 mm. 

**Figure 2:**
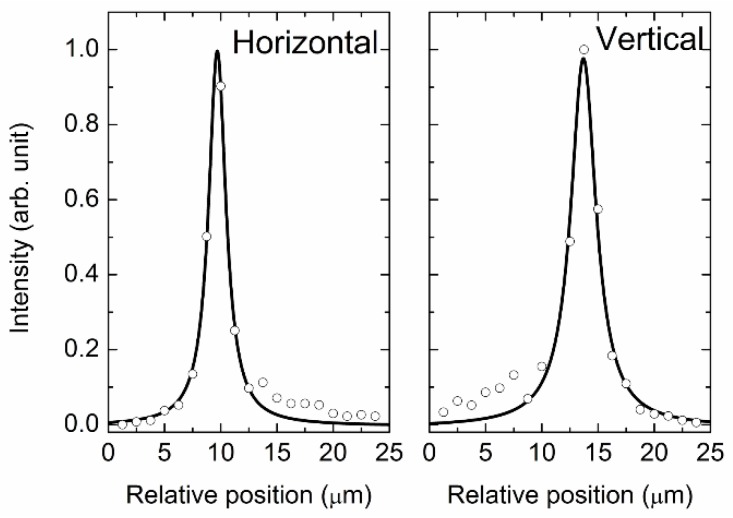
Light intensity of the focal unmonochromatized SR beam against the horizontal and vertical positions at the focal position. The collected data (open circle) were fitted by a Lorentz function (solid line).

The light intensity of the focal unmonochromatized SR beam against the horizontal and vertical directions was measured by the PMT (R6836; Hamamatsu Photonics, Shizuoka, Japan) using a knife-edge method, and the data were fitted by the Lorentz function, as shown in Figure 2. The overall spot size was ca. 25 × 25 μm^2^ at the focal position, and the full width at half-maximum of the beam intensities was 1.9 μm (horizontal) and 2.7 μm (vertical), indicating that the effective spot size would be much smaller than 25 × 25 μm^2^. The spot size without the SO was ca. 6 × 6 mm^2^, and thus it can be observed that the size was reduced down to 1/240 or less.

**Figure 3:**
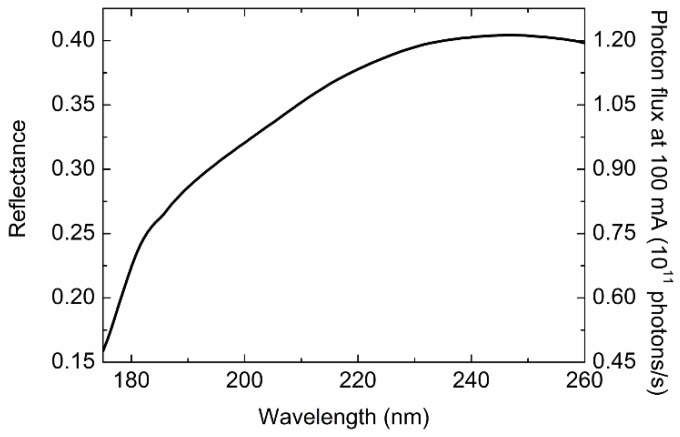
Reflectance of the SO (left axis) and photon flux (normalized at 100 mA electron beam current) after passing through the SO (right axis) in the wavelength region of 175–260 nm.

Figure 3 shows the reflectance of the SO in the wavelength region of 175–260 nm. The beam intensity was reduced down to 16–40% owing, presumably, to the bi-reflection in the SO system. The photon flux after the SO (Figure 3) was smaller than that of most of the other SRCD beamlines [6,7]. Nevertheless, the beam intensity was enough to measure the CD spectra, as shown in Section 2.4 and Section 2.5. The intensity of the focal beam would be improved by using optimized mirrors with high reflectance in the UV–VUV region.

### 2.2. Determination of the Sample Position

Although the sample should be set at the focal position to reduce the required sample volume, great care should be taken regarding the radiation damage of samples such as biomolecules, because the photon flux density, which is the photon flux divided by the area of the beam spot, is the highest at the focal position. Indeed, in this study, the photon flux density at the focal position was 2 × 10^14^ photons·s^−1^·mm^−2^ at 200 nm, which is much higher than that of other SRCD beamlines [6,7]; this is due to the small spot area (ca. 5 × 10^−4^ mm^2^), although the total flux is low, as mentioned above. Radiation damage during CD measurements is often observed at high-flux-density SRCD beamlines [8,9]. For example, at the CD12 beamline of SRS in the U.K., the CD spectrum of myoglobin differed between the first and the second spectra, i.e., denaturation was induced by radiation damage within only 10 min [10], even though the photon flux density of the CD12 beamline was only 1% that of the focal position of the SO system used in this study. These results indicate that protein samples placed at the focal position of this system would be immediately decomposed.

Therefore, the sample position was carefully adjusted, comparing the flux density to that of other SRCD beamlines. As a result, the sample position was set at about 4 mm behind the focal position. At this point, the defocused spot area was about 1 mm^2^, and the photon flux density was 1 × 10^11^ photons·s^−1^·mm^−2^ at 200 nm, which is comparable to the flux density threshold value for denaturation proposed by Miles et al. [9]. Evaluation of the radiation damage is discussed in Section 2.4.1.

### 2.3. Small-Capacity Sample Cell

**Figure 4:**
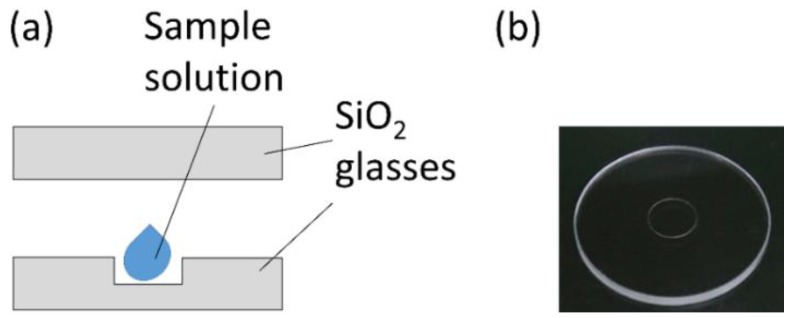
(**a**) A schematic view of the developed cell. (**b**) A photograph of the counterbored glass.

A schematic view of the developed cell is shown in Figure 4. It was composed of two synthetic silica glasses with a thickness of 1 mm and an outer diameter of 20 mm. One of the glasses was flat (Ohyo Koken Kogyo, Tokyo, Japan) and the other had a counterbore hole 5 mm in diameter. The depth of the counterbore hole (15 μm: ATOCK, Ibaraki, Japan and 60 μm: Kyokuei-Kenma, Tokyo, Japan) corresponds to the path length of the cell. The hole was filled with the sample solution and covered with the flat glass. The capacities of the counterbore holes with 15 and 60 μm depths are 0.29 and 1.2 μL, respectively, and are comparable with those of CaF_2_ micro cells from Hellma Optics (Jena, Germany) [11].

Basically, the sample volume injected into the cell was much larger in practice to avoid foaming, although the volumes would depend on the types of solutions and skill of users. The counterbored glasses with depths of 15 μm and 60 μm developed in this study required 1.5 and 2.0 μL sample solution, respectively. The sample volume described here was reduced down to 1/10 or less as compared to that required by the pre-existing cells used at the BL-12 of HiSOR (15 and 20 μL for 10 and 50 μm pathlength cells, respectively) [12] and the commercial micro-volume cells (~20 μL for 10 μm pathlength cell) [13,14,15] for laboratory-based CD spectrophotometers. On the other hand, the sample volume of the developed cells was comparable to those required by the CaF_2_ micro cells [11] (2 μL and 3 μL for 12 and 13.3 μm pathlength cells, respectively, in recent reports using other SRCD beamlines [16,17]) owing to the similar capacities of the cells. From this point of view, the benefit of the SO may seem low for users of other SRCD beamlines. However, since the area of the counterbored hole (ca. 20 mm^2^) used in this study is much larger than the size of the defocused beam (ca. 1 mm^2^) given by the SO system, the volumes can be reduced further by adopting a smaller counterbored hole (estimated sample volume: 0.04 μL for 10 μm pathlength and 1 mm^2^ sample area). Then, the effect of the SO system on the volume reduction would be also obtained in other SRCD beamlines.

### 2.4. Performance of CD Measurement Using the SO

#### 2.4.1. Radiation Damage to Protein Sample

**Figure 5:**
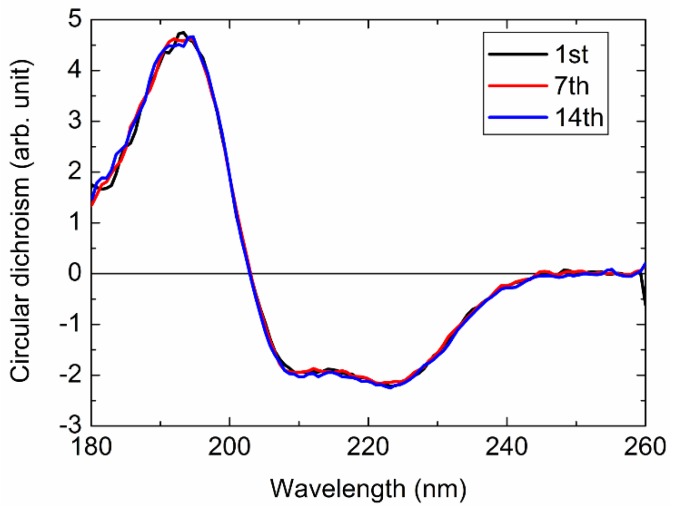
CD spectra of myoglobin obtained by consecutive scans for 1 h. 1st scan: black, 7th scan (0.5 h later): red, and 14th scan (1 h later): blue.

Figure 5 shows the CD spectra of myoglobin of the 1st, 7th (0.5 h later), and 14th scan (1 h later). These spectra were in good agreement with one another. Thus, it could be concluded that the radiation damage during CD measurements would be negligible for at least 1 h within this wavelength region. The measurement time for each sample is usually shorter than 1 h at the BL-12 of HiSOR; therefore, it was concluded that additional treatments to prevent radiation damage, such as closing the slits, were unnecessary as long as the CD measurements were performed at this defocus point.

#### 2.4.2. Distortion of CD Spectrum by the SO

**Figure 6:**
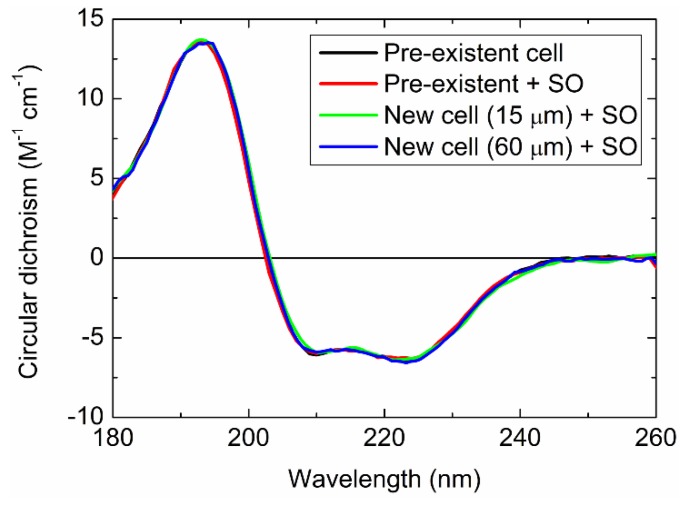
CD spectra of myoglobin measured using the pre-existent cell (black), pre-existent cell and SO (red), new cell (15 μm path length) and SO (green), and new cell (60 μm path length) and SO (blue).

To confirm whether the SO and/or new cells cause distortion in the CD spectra, the CD spectra of myoglobin were measured using (1) the pre-existent sample cell, (2) pre-existent cell and SO, (3) new cell (15 μm path length) and SO, and (4) new cell (60 μm path length) and SO, as shown in Figure 6. These spectra demonstrated good agreement with one another in the wavelength region from 180 to 260 nm. Hence, the use of the new sample cells with the SO would cause no distortion in the CD spectra, at least within this wavelength range. Linear dichroism and birefringence (LD and LB, respectively) exhibited by the cells and the SO would also be negligible within this range.

### 2.5. Structural Analysis of H3K36me3

Finally, the CD spectrum of H3K36me3 was measured using the SO system and small-capacity sample cell.

Knowledge of post-translational modifications (such as methylation) of histone proteins, induced during the DNA damage repair process in eukaryotic cells, has increased over time [18,19,20,21,22,23,24,25]. For example, it is assumed that H3K36me3 is linked to DNA repair in transcriptionally active regions, and it is thought the DNA repair processes involve drastic structural alterations of chromatin, namely complex of DNA and histone proteins, induced by the post-translational modifications of histones [21,22,26,27]. However, experimental and theoretical studies on the structural changes of histone, induced by modifications or DNA damage repair processes, are not so abundant [17,28,29,30,31,32,33,34,35,36,37]. To precisely understand the mechanism of chromatin structural remodeling, cyclopedic studies of modification-induced structural changes of histones would be necessary.

#### 2.5.1. CD Spectrum of H3K36me3

**Figure 7:**
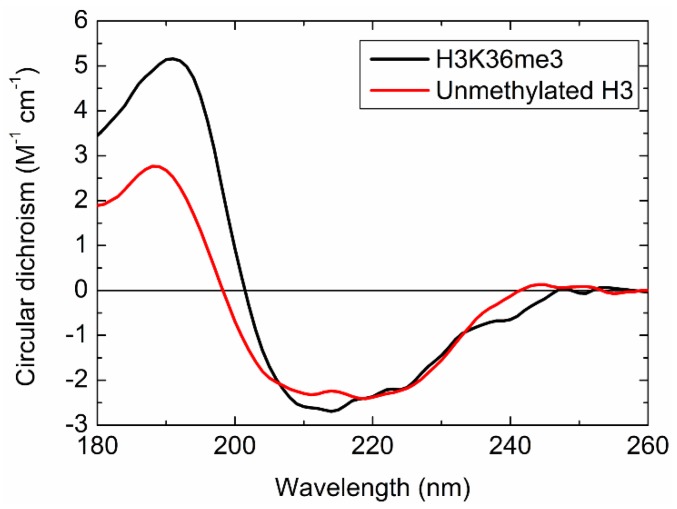
CD spectra of H3K36me3 (black) and unmethylated H3 (red), reproduced from literature data [36].

Figure 7 shows the CD spectrum of H3K36me3. The spectrum of unmethylated H3, which is reproduced from the data of ref. [36], is also shown in this figure for comparison. Unmethylated H3 exhibited a positive peak around 190 nm and negative peaks around 210 and 220 nm; these were characteristic CD peaks of α-helix structures [38]. H3K36me3 showed a spectral shape similar to that of unmethylated H3, but with largely different peak intensities and positions. Since CD spectra reflect protein conformations, the spectral differences show that the trimethylation of H3 lysine-36 residue induced structural alteration.

#### 2.5.2. Secondary Structural Contents of H3K36me3

The secondary structural contents of H3K36me3 and unmethylated H3 [36] are listed in Table 1, where each content is 100% normalized. As shown in Table 1, the secondary structural contents were increased in the helix (25.0 ± 1.2% → 35.6 ± 1.3%) by the trimethylations. On the other hand, unordered structures were decreased (32.7 ± 1.7% → 23.3 ± 2.8%). The contents of the β-strand and turn structures were the same within the error range. For the trimethylations of lysine-4 or lysine-9 residues of H3 (H3K4me3 and H3K9me3, respectively), a decrement in the content of helix structures and an increment of β-strand structures were observed [36,37], showing that the structural alterations observed in H3K4me3 and H3K9me3 were quite different from those of H3K36me3. These results suggest that the structural changes induced by post-translational trimethylations are not uniform, but are rich in variation depending on the sites of methylation. The various structural changes induced by post-translational modifications may be strongly related to the regulation of cellular functions.

#### 2.5.3. Predicted Positions of α-Helices and β-Strands

**Figure 8:**
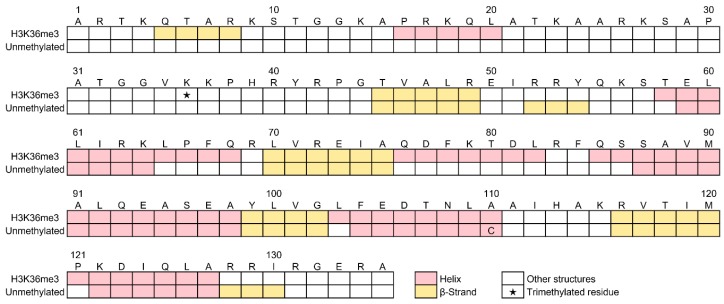
Sequence-based secondary structures of H3K36me3 and unmethylated H3 predicted by the VUVCD-neural network (NN) method. The data of unmethylated H3 were reproduced from a previous paper [36]. The helix, β-strand, and other structures are shown in pink, yellow, and white rectangles, respectively. The star in the rectangle shows the trimethylated residue. The 110th residue of unmethylated H3 is cysteine (C) and is different from that of H3K36me3 (alanine; A).

Figure 8 shows the predicted secondary structural sequences of H3K36me3 and unmethylated H3 [36]. Remarkable results in H3K36me3 were the β-strand formation in the 5th–8th residues and helix formations in the 16th–20th residues. These residues were located in the *N*-terminal tail of H3. This tail region strongly interacts with various histone-binding proteins, such as enzymes, in cells [18,19,20,21,22,23,24,25,26,27]. Thus, the structural alterations in the *N*-terminal tail induced by the modification(s) would affect the reactivity. In addition, a newly formed helix segment (76th–82nd), some elongated helix segments (58th–68th and 85th–98th), and decrement of β-strand segments (52nd–54th and 128th–130th) were also predicted other than in the region of the *N*-terminal tail. These results show that the structural alterations of isolated H3 are not limited to the region around the methylation site. To investigate the interactions between the histone-binding proteins and H3K36me3 [39,40,41,42] or other (un)modified histone proteins in vitro, short peptides in the vicinity of the modification site(s) (ca. 10–20 residues) are often used. However, the prediction implies that the residues distant from the modification site(s) should also be considered. Indeed, it is known that the catalytic activities of lysine-specific demethylase 1 and 2 (LSD1 and LSD2, respectively) depend on the peptide-length of lysine-4 methylated H3; the first 21 residues, at least, of lysine-4 mono- or dimethylated H3 are required for effective demethylation, though only the first 12–16 residues seem to contact the binding site of LSD1 and LSD2 [43,44,45,46]. It is also known that the binding affinity between LSD1 and full-length unmethylated H3 is nearly 100-fold higher than that between LSD1 and unmethylated H3 peptides (first 21 residues) [47].

It seems that the optimum peptide length for mimicking cellular histones and/or chromatin has not been sufficiently investigated. Systematic studies on the optimum peptide length should be carried out. 

## 3. Materials and Methods 

### 3.1. Sample Preparation

A reagent of myoglobin from a horse heart (purity > 90%) was purchased from Sigma-Aldrich (St. Louis, MO, USA) and used without further purification. The reagent was dissolved in Milli-Q water, and the concentration of the sample was about 5 μg/μL.

Recombinant *Xenopus laevis* H3K36me3 (purity > 98%), synthesized using methylated lysine analog technology [48], was purchased from Active Motif (Carlsbad, CA, USA). The reagent was dissolved in a 25 mM sodium phosphate buffer supplemented by 250 mM sodium fluoride (pH = 8.6 at 25 °C) and used without further purification. It is known that this solvent condition can prevent the aggregation of methylated H3 proteins [37]. The concentration of H3K36me3 was 1 μg/μL.

### 3.2. CD Measurements

All CD spectra were measured between 180 and 260 nm at 25 °C, using the VUVCD spectrophotometer at HiSOR [5]. The consecutive scans of myoglobin described in Section 2.4.1 were carried out using the new cell with a path length of 60 μm and the SO. For evaluation of the spectral distortion, CD spectra of myoglobin were measured five times and averaged in each condition described in Section 2.4.2. The CD spectrum of the solvent, which should be zero under ideal conditions, was also measured as a baseline. This baseline was subtracted from the CD spectra of the samples to remove artificial CD signals that might have originated from the optical systems, cells, etc.

The CD spectra of H3K36me3 were measured twice (5 scans/measurement), using the SO and the new cell with a 15 μm path length, in a similar manner. The data of H3K36me3 and the baseline are deposited as a supplementary information in this journal.

### 3.3. Analysis of Secondary Structures

We used the SELCON3 program [49,50] based on the reference proteins measured at HiSOR [51,52] to analyze the contents of helix, β-strand, turn, and unordered structures and numbers of segments of helix and β-strand in H3K36me3. The program and the dataset were selected for the following reasons: (i) the program can successfully provide the numbers of helix and β-strand segments which are necessary for estimating those positions (see detail in below), and (ii) the dataset of the reference proteins was obtained using the same CD instrument at HiSOR and it allows us to avoid any inaccuracy and/or ambiguity which might originate from the usage of different CD instruments [53]. We also analyzed the secondary structure contents of H3K36me3 using CONTIN/LL program [54,55] and a dataset SP29 provided in CDPro software package [50] for confirmation and obtained comparable results. However, the results obtained here (e.g., for the helix content) are still controversial because other programs and datasets might provide the different results as reported in a previous paper [56]. Although SELCON3 program based on the 31 reference proteins was used in this study by the reasons mentioned above, the usage of larger reference dataset such as SP175 (more than 70 proteins) [57] and the latest program such as BeStSel [58] should be considered in future. The SELCON3 program was applied over the wavelength range of 185–260 nm. It is noted that helix content include the α-helix and 3_10_-helix structures and the content of unordered structures includes bend, π-helix, and β-bridge structures.

The positions of the helices and β-strands in H3K36me3 were estimated by a neural network (NN) method based on the CD spectroscopic results, which is termed the VUVCD-NN combination method. In this prediction, methylated residue was regarded as normal lysine residues. The computational protocol is described elsewhere [59]. We used a NN algorithm that predicts the positions of secondary structures by using evolutionary sequence information based on the position-specific scoring matrices generated by the PSI-BLAST algorithm [60], referring to the numbers of amino acid residues of helices and β-strands determined by CD spectroscopy and SELCON3 analyses (*N*_h_ and *N*_β_, respectively) and the number of segments of helices and β-strands (*n*_h_ and *n*_β_, respectively). The *N*_h_ and *N*_β_ were calculated by multiplying the total number of amino acid residues of H3K36me3 (*N* = 135) with the secondary structural contents of helix and β-strand structures, respectively. *n*_h_ and *n*_β_ were calculated by the following equations [49]:

*n*_h_ = h_D_*N*/4 (1)

and

*n*_β_ = β_D_*N*/2 (2)

where h_D_ and β_D_ are the contents of the helix and β-strand structures, respectively, in distorted regions. We assumed that the distorted region corresponds to two residues at each end of the helix structure or one residue at each end of the β-strand structure. In this work, h_D_ and β_D_ were 17.7 ± 1.1 and 7.2 ± 0.7%, respectively. The turn and unordered structures estimated by the SELCON3 analysis were classified as other structures. It is known that the accuracy of the VUVCD-NN combination method is about 75% for 30 reference proteins [59].

## 4. Conclusions

The beam size at the focal position was reduced down to 1/240 (6 × 6 mm^2^ → 25 × 25 μm^2^) or less using the SO. The use of the SO and developed small-capacity sample cell allowed the sample volume to be reduced to 1/10 (20 μL → 2 μL for a 60 μm path-length cell and 15 μL → 1.5 μL for a 15 μm path-length cell). Each required volume is one of the smallest among those for similar path-length cells, to our knowledge. It could be reduced more when adopting a smaller sample area and path-length cell. The high-flux-density beam at the focal position is very attractive for CD measurements under the flow condition, such as time-resolved CD measurements, because the exposure time becomes quite short (in the millisecond time range) [3], making the radiation damage negligible. The system developed in this study can be easily available to other SR-based and commercial CD spectrophotometers; thus, it is expected to contribute to advances in CD spectroscopic science.

As an application of this system, the CD spectrum of a scarce protein (H3K36me3) was measured and its secondary structures were analyzed. From the comparison between H3K36me3 and unmethylated H3, the increment of helix structures and the decrement of unordered structures were clearly observed. Post-translational methylation can induce various structural alterations of histones, depending on the methylation sites. Those structural alterations may contribute to regulating cellular functions such as DNA damage repair systems. Cyclopedic structural analyses of modified histones are important for understanding the role of the modification-induced structural changes of histones. Obtaining or refining the proteins involved in the DNA damage repair system is generally difficult; thus, this system will contribute to easing the challenge of structural analyses of such proteins.

## Figures and Tables

**Table 1:** The secondary structural contents of H3K36me3 and unmethylated H3, obtained using the SELCON3 program.

Structure Content (%)	H3K36me3	Unmethylated H3 ^1^
Helix	35.6 ± 1.3	25.0 ± 1.2
β-strand	18.7 ± 2.5	21.3 ± 1.5
Turn	22.3 ± 0.9	21.1 ± 1.0
Unordered	23.3 ± 2.8	32.7 ± 1.7

^1^ Reproduced from ref. [36].

## Supplementary Materials

The following is available online, spectral data of H3K36me3 and the baseline.
